# *p53* codon 72 polymorphism and Hematological Cancer Risk: An Update Meta-Analysis

**DOI:** 10.1371/journal.pone.0045820

**Published:** 2012-09-24

**Authors:** Yu Weng, Liqin Lu, Guorong Yuan, Jing Guo, Zhizhong Zhang, Xinyou Xie, Guangdi Chen, Jun Zhang

**Affiliations:** 1 Department of Clinical Laboratory, Zhejiang Univerisity School of Medicine, Sir Run Run Shaw Hospital, Hangzhou, China; 2 Department of Oncology, Zhejiang Provincial People’s Hospital, Hangzhou, China; 3 Department of Public Health, Institute of Environmental Health, Zhejiang Univerisity School of Medicine, Hangzhou, China; 4 Department of Neurology, School of Medicine, Nanjing University, Jinling Hospital, Nanjing, China; University of North Carolina at Chapel Hill, United States of America

## Abstract

**Background:**

Previous studies on the association of *p53* codon 72 (Arg72Pro) polymorphism with hematological malignancies risk have produced conflicting results. The purpose of this meta-analysis is to define the effect of *p53* Arg72Pro polymorphism on hematological malignancies risk.

**Methodology/Principal Findings:**

Through searching PubMed databases (or hand searching) up to April 2012 using the following MeSH terms and keywords: “p53”, “codon 72” “polymorphism” and “leukemia”, or “lymphoma”, or “myeloma”, thirteen were identified as eligible articles in this meta-analysis for *p53* Arg72Pro polymorphism (2,731 cases and 7, 356 controls), including nine studies on leukemia (1,266 cases and 4, 474 controls), three studies on lymphoma (1,359 cases and 2,652 controls), and one study on myeloma. The overall results suggested that *p53* Arg72Pro polymorphism was not associated with hematological malignancies risk. In stratified analyses, significantly increased non-Hodgkin lymphomas risk was found in *p53* Arg72Pro polymorphism heterozygote model (Arg/Pro *vs*. Arg/Arg: OR = 1.18, 95%CI: 1.02–1.35) and dominant model (Arg/Pro+Pro/Pro *vs*. Arg/Arg: OR = 1.18, 95%CI: 1.03–1.34), but no significant association was found between leukemia risk and *p53* Arg72Pro polymorphism. Further studies showed no association between leukemia risk and *p53* Arg72Pro polymorphism when stratified in subtypes of leukemias, ethnicities and sources of controls.

**Conclusions/Significance:**

This meta-analysis indicates that the *p53* Arg72Pro polymorphism may contribute to susceptibility to non-Hodgkin lymphomas.

## Introduction

Hematological malignancies derived from either of the two major blood cell lineages: myeloid and lymphoid cell lines, include leukemias, lymphomas, myeloma, myelodysplastic syndromes and myeloproliferative diseases. Lymphomas, lymphocytic leukemias, and myelomas are from the lymphoid line, while acute and chronic myelogenous leukemia, myelodyplastic syndromes and myeloproliferative diseases are myeloid in origin. Generally, the overall incidence of hematological malignancies appears to be rising in Western countries but it is very difficult to describe on their epidemiological behavior in a consistent way [1]. In the USA, the number of estimated new cases of hematological malignancies in 2011 was 140,310 and it was predicted to have 53,010 deaths due to hematological malignancies [2]. Hematological malignancies are very heterogeneous diseases with respect to clinical features and acquired genetic alterations. The etiology of hematological malignancies appears to be multifactorial, including the inherited mutations in DNA, and exposure to ionizing radiation, or to chemicals like benzene or cytotoxic therapy. Exposure to these carcinogens may cause DNA damage at the level of hematopoietic progenitors and develop hematological malignancies; however, the majority of cases likely involve genetic variations with a high-risk phenotype [3]. These gene-gene interactions, as well as their interplay with lifestyle-related factors and environmental agents, may be major determinants in hematological malignancy susceptibility [4].

The tumor suppressor p53 plays a pivotal role in response to genotoxic insults from endogenous or environmental agents by orchestrating a diversity of pathways from activation of cell signaling transduction, transcriptional responses, DNA repair to regulation of cell cycle progression and apoptosis [5]. Although *p53* mutations are commonly found in different cancers and thought to be associated with carcinogenesis [6], [7], [8], polymorphisms in *p53* seem to have a modest effect on cell phenotype, leading to different patterns of cancer susceptibility [9], [10]. The *p53* gene locates on chromosome 17p13 and contains 11 exons. The common *p53* polymorphisms include *p53* codon 72 (c.215C>G; p.R72P; rs1042522), deletion of 16 bp in intron 3 (c.96+41_96+56del16; rs17878362) and IVS6+62A>G (c.672+62A>G; rs1625895) polymorphisms [10]. Among them, *p53* codon 72 (Arg72Pro) polymorphism is most widely studied in different cancers [11].

The *p53* codon 72 polymorphism is located in exon 4 with CGC to CCC transition, leading to an arginine-to-proline amino-acid substitution in amino-acid position 72 [10]. Laboratory studies have demonstrated the Arg variant is more potent in apoptosis induction whereas the Pro variant is better in inducing cell cycle arrest and DNA damage repair [12], [13], [14]. Recently, many of epidemiological studies have examined the association between *p53* Arg72Pro polymorphism and hematological malignancies risk, however, these studies revealed an inconsistent conclusion, probably due to the relatively small size [11], [15], [16], [17]. Therefore, a meta-analysis was performed from all eligible studies to evaluate the association between *p53* Arg72Pro polymorphism and hematological malignancies risk in this study.

## Materials and Methods

### Identification and Eligibility of Relevant Studies

To identify all articles that examined the association of *p53* codon 72 polymorphism with hematological malignancies, we conducted a literature search in the PubMed databases up to April 2012 using the following MeSH terms and keywords: “p53”, “codon 72” “polymorphism” and “leukemia”, or “lymphoma”, or “myeloma”. Additional studies were identified by a hand search from references of original studies or review articles on this topic. Eligible studies included in this meta-analysis had to meet the following criteria: (a) an unrelated case-control study, if studies had partly overlapped subjects, only the one with a larger sample size was selected, (b) available genotype frequency, (c) sufficient published data for estimating an odds ratio (OR) with 95% confidence interval (CI) and (d) the genotype frequencies in the control group were consistent with Hardy-Weinberg equilibrium (HWE).

### Data Extraction

Two investigators independently extracted data and reached a consensus on all of the items. The following information was extracted from each study: first author, year of publication, country of origin, ethnicity, number of cases and controls, genotype frequency for cases and controls, characteristics for cases, sources of DNA and genotyping methods. Different ethnicity descents were categorized as Asian and Caucasian.

### Statistical Analysis

Hardy-Weinberg equilibrium (HWE) was tested by the chi-square test. Crude ORs with 95% CIs were used to assess the strength of association between the *p53* Arg72Pro polymorphism and hematological malignancy risk. We first estimated the risks of the Arp/Pro and Pro/Pro genotypes on hematological malignancies, compared with the reference Arg/Arg homozygote, and then evaluated the risks of (Arp/Pro+Pro/Pro *vs*. Arg/Arg) and (Pro/Pro *vs*. Arg/Arg + Arp/Pro) on hematological malignancies, assuming dominant and recessive effects of the variant Pro/Pro allele, respectively [11], [18], [19].

Stratified analyses were also performed by types of hematological malignancies, ethnicities and sources of controls. Potential heterogeneity was checked by the χ^2^-based Q-test. The summary OR estimate of each study was calculated by the random-effects model (the DerSimonian and Laird method).

Publication bias was investigated by funnel plot, and an asymmetric plot suggested possible publication bias. The funnel plot asymmetry was assessed by Egger’s linear regression test. The *t* test was performed to determine the significance of the asymmetry, and a *P* value of <0.05 was considered a significant publication bias. All analyses were done with Stata software (version 11.0; StataCorp LP, College Station, TX), using two-sided *P* values.

## Results

### Characteristics of Studies

**Figure 1:**
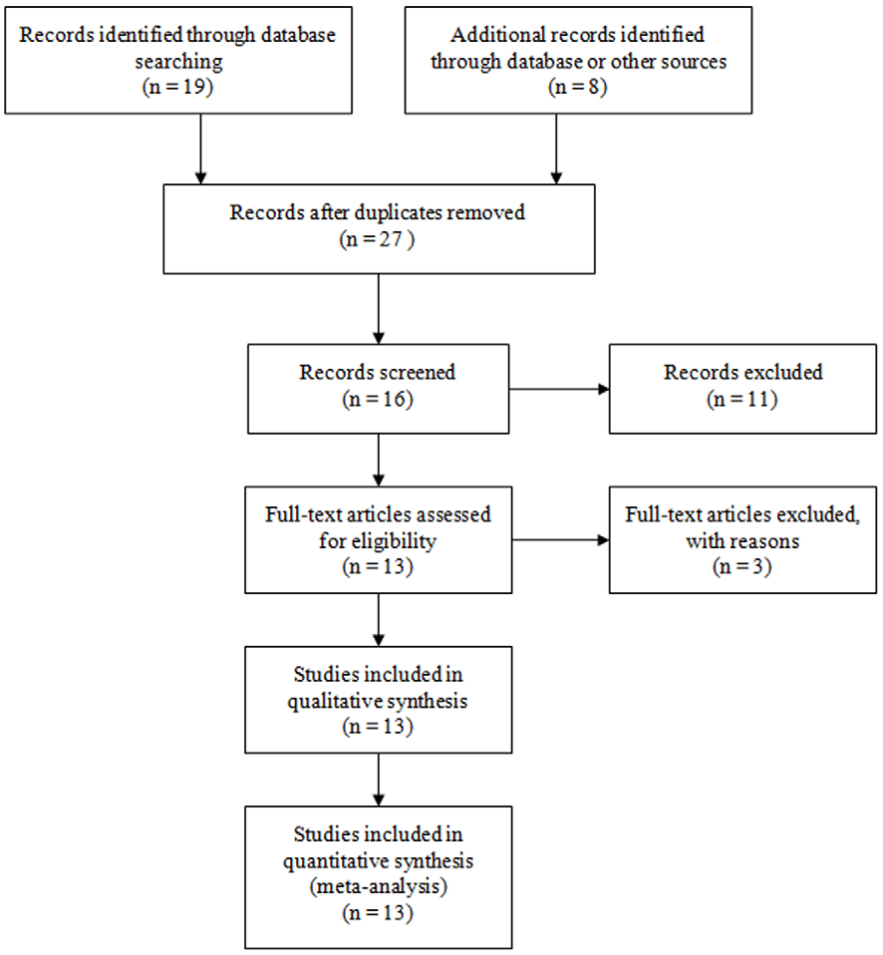
Flow diagram of studies identification.

Nineteen abstracts were retrieved through the search “p53”, “codon 72”, “polymorphism” and “leukemia”, and eight studies were identified as eligible studies. Out of the nineteen, seven studies were excluded given that they have not included controls, did not report genotype frequency for controls in their study designs, or reported other diseases [20], [21], [22], [23], [24], [25], [26], one article was review [27], and three studies were *in vitro* cell biology studies [28], [29], [30]. We also included eligible study with hand searching [31]. By searching “p53”, “polymorphism” and “lymphoma” or “myeloma”, and a hand search from references of original studies or review articles, we included another seven articles [16], [17], [32], [33], [34], [35], [36]. The genotype distributions among the controls of all studies were in agreement with Hardy-Weinberg equilibrium except for three studies [16], [33], [36](Figure 1). As a result, a total of thirteen studies met the inclusion criteria and were identified as eligible articles with 2,711 cases and 7,356 controls, including nine studies of leukemia [15], [31], [37], [38], [39], [40], [41], [42], [43], three studies of lymphoma [17], [32], [34] and one study of myeloma [35]. The selected study characteristics were listed in Table 1. The patients’ demographic characteristics and *p53* genotype distribution were listed in Table S1 and S2.

**Table 1:** Characteristics of literatures included in the meta-analysis.

Author	Year	Origin	Ethnicity	Sample size (case/control)	HWE	MAF	Design	Genotype
Leukemia								
Nakano Y	2000	Japan	Asian	200/188	0.77	0.43	PB	SSCP
Bergamaschi G	2004	Italy	Caucasian	96/174	0.82	0.22	PB	PCR-RFLP
Takeuchi S	2005	Japan	Asian	87/89	0.19	0.43	HB	PCR-RFLP
Kochethu G	2006	UK	Caucasian	203/97	0.50	0.34	PB	PCR-RFLP
Phang BH	2008	China	Asian	44/160	0.33	0.43	PB	PCR-RFLP
Ellis NA	2008	USA/UK	Caucasian	171/3022	0.85	0.25	PB	Taqman/PCR-RFLP
Xiong X	2009	China	Asian	231/128	1.00	0.45	HB	PCR-RFLP
Do TN	2009	US	Caucasian	114/414	0.90	0.25	PB	Taqman
Chauhan PS	2011	India	Asian	120/202	0.09	0.49	PB	PCR-RFLP
Lymphomas								
Hishida A	2004	Japan	Asian	103/440	0.84	0.35	HB	Allele PCR
Bittenbring J	2008	Germany	Caucasian	311/512	0.81	0.25	HB	Allele PCR
Kim HN	2010	Korea	Asian	945/1700	0.52	0.34	PB	Taqman
Myeloma								
Ortega MM	2007	Brazil	Caucasian	106/230	0.09	0.37	HB	PCR-RFLP

Abbreviations: HB, Hospital based controls; PB, population based controls; PCR, Polymerase chain reaction; PCR-RFLP, Polymerase chain reaction- restriction fragment length polymorphism; SSCP, Single-Strand Conformation Polymorphism; HWE, Hardy-Weinberg equilibrium; MAF, minor allele frequency.

The genotyping for *p53* codon 72 polymorphism was performed using polymerase chain reaction (PCR), polymerase chain reaction- restriction fragment length polymorphism (PCR-RFLP), Taqman PCR, or single-strand conformation polymorphism (SSCP) analyses on the genomic DNA from the human blood samples. For ethnic distribution, there were seven studies of Caucasian descent, and six of Asian origin. For the nine studies on leukemia, there were five studies on acute myeloid leukemia (AML) and four studies on others including acute lymphoblastic leukemia (ALL), chronic lymphoblastic leukemia (CLL) and chronic myeloid leukemia (CML). As to ethnic distribution of the leukemia patients, there were five studies of Asians and four studies of Caucasians.

**Table 2:** Meta-analysis of the *p53* codon 72 Arg>Pro polymorphism on hematological malignancy risk.

Variables	n^a^	Arg/Pro vs. Arg/Arg		Pro/Pro vs. Arg/Arg		Arg/Pro + Pro/Pro vs. Arg/Arg (dominant)		Pro/Pro vs. Arg/Arg + Arg/Pro (recessive)	
		OR (95% CI)	*P* b	OR (95% CI)	*P* b	OR (95% CI)	*P* b	OR (95% CI)	*P* b
Total	13	1.08(0.95–1.24)	0.199	1.10(0.83–1.45)	0.004	1.08(0.93–1.26)	0.033	1.05(0.83–1.33)	0.026
Types									
Leukemia	9	1.03(0.83–1.27)	0.100	1.10(0.71–1.72)	0.001	1.04(0.81–1.34)	0.010	1.07(0.75–1.53)	0.010
Lymphoma	3	1.18(1.02–1.35)	0.797	1.12(0.80–1.55)	0.257	1.18(1.03–1.34)	0.719	1.02(0.74–1.40)	0.249
Myeloma	1								
Ethnicities									
Asian	7	1.11(0.92–1.34)	0.235	0.99(0.73–1.33)	0.122	1.07(0.86–1.33)	0.106	1.00(0.83–1.21)	0.389
European	6	1.05(0.85–1.29)	0.190	1.32 (0.74–2.35)	0.003	1.09(0.85–1.40)	0.037	1.31(0.79–2.17)	0.010
Source of controls									
Hospital based	5	1.00(0.84–1.18)	0.099	0.98(0.73–1.33)	0.048	0.99(0.82–1.19)	0.027	1.00(0.80–1.26)	0.209
Population based	8	1.02(0.84–1.25)	0.069	1.12(0.73–1.72)	0.001	1.04(0.82–1.31)	0.006	1.11(0.78–1.58)	0.007

a Number of comparisons.

b *P* value of Q-test for heterogeneity test.

**Table 3:** Meta-analysis of the *p53* codon 72 Arg>Pro polymorphism on leukemia risk.

Variables	n^a^	Arg/Pro vs. Arg/Arg		Pro/Pro vs. Arg/Arg		Arg/Pro + Pro/Pro vs. Arg/Arg (dominant)		Pro/Pro vs. Arg/Arg + Arg/Pro (recessive)	
		OR (95% CI)	*P* b	OR (95% CI)	*P* b	OR (95% CI)	*P* b	OR (95% CI)	*P* b
Leukemia	9	1.03(0.83–1.27)	0.100	1.10(0.71–1.72)	0.001	1.04(0.81–1.34)	0.010	1.07(0.75–1.53)	0.010
Ethnicities									
Asian	5	1.01(0.74–1.39)	0.186	0.84(0.57–1.26)	0.205	0.97(0.70–1.34)	0.119	0.85(0.64–1.12)	0.569
Caucasian	4	1.34(0.73–1.46)	0.070	1.57(0.68–3.61)	0.003	1.12(0.74–1.69)	0.009	1.55(0.77–3.11)	0.014
Source of controls									
Hospital based	2	1.28(0.85–1.92)	0.348	1.16(0.59–2.28)	0.185	1.22(0.76–1.97)	0.214	1.03(0.67–1.59)	0.353
Population based	7	0.98(0.76–1.26)	0.086	1.10(0.62–1.94)	0.001	1.00(0.74–1.35)	0.007	1.10(0.69–1.77)	0.004
Types									
AML	5	1.03(0.78–1.35)	0.180	0.89(0.60–1.30)	0.197	0.99(0.75–1.32)	0.108	0.88(0.67–1.16)	0.559
Others c	4	1.06(0.67–1.53)	0.069	1.49(0.60–3.71)	0.002	1.09(0.66–1.79)	0.008	1.48(0.69–3.17)	0.007

a Number of comparisons.

b *P* value of Q-test for heterogeneity test.

c Others include acute lymphoblastic leukemia, chronic lymphocytic Leukemia, chronic myeloid leukemia.

Abbreviations: AML, Acute myeloid leukemia.

### Quantitative Synthesis

**Figure 2:**
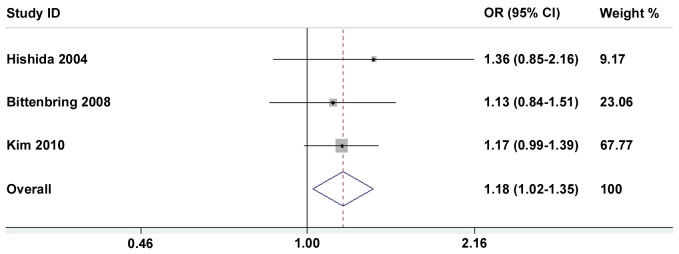
Forest plots of heterozygote model (Arg/Pro vs. Arg/Arg) in different subgroups. The squares and horizontal lines correspond to OR and 95% CI of specific study, and the area of squares reflects study weight (inverse of the variance). The diamond represents the pooled OR and its 95% CI.

**Figure 3:**
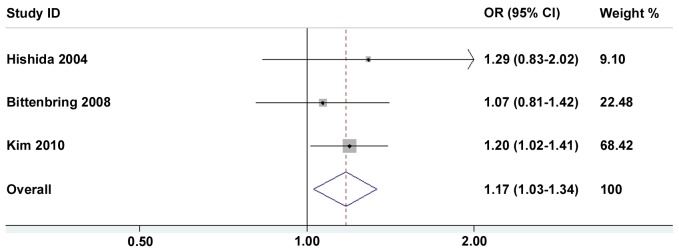
Forest plots of dominant model (Arg/Pro + Pro/Pro vs. Arg/Arg) in different subgroups. The squares and horizontal lines correspond to OR and 95% CI of specific study, and the area of squares reflects study weight (inverse of the variance). The diamond represents the pooled OR and its 95% CI.

Tables 2 and 3 present in detail the results of the meta-analysis. By pooling all the studies, the *p53* Arg72Pro polymorphism was not associated with a hematological malignancies risk, and this negative association maintained in some subgroup analyses such as ethnicities and sources of controls (Table 2). When stratified by hematological malignancies types, no association was found between *p53* Arg72Pro polymorphism and leukemia risk (1,266 cases and 4474 controls) in all four models (Table 2). However, *p53* Arg72Pro polymorphism heterozygote (Arg72Pro) was significantly correlated increased lymphomas risk (Arg/Pro *vs*. Arg/Arg: OR = 1.18, 95%CI: 1.02–1.35) (Figure 2), and this association was further confirmed in dominant model (Arg/Pro+Pro/Pro *vs*. Arg/Arg: OR = 1.18, 95%CI: 1.03–1.34) (Figure 3). Actually, all cases included in these three eligible studies on lymphomas were non-Hodgkin lymphoma patients (NHL) (1,359 cases and 2,652 controls). Thus, our data suggest an association between *p53* Arg72Pro polymorphism and NHL risk.

We next analyzed the association between *p53* Arg72Pro polymorphism and leukemia risk when stratified by the ethnicities, sources of controls, and leukemia types. The results showed that the *p53* Arg72Pro polymorphism was not associated with leukemia either in Asians or in Caucasians, and this negative association maintained in other subgroup analyses such as leukemia types and sources of controls (Table 3).

### Publication Bias

Begg’s funnel plot and Egger’s test were performed to assess the publication bias of literatures. The shapes of the funnel plots did not reveal any evidence of obvious asymmetry (Data not shown). Then, the Egger’s test was used to provide statistical evidence of funnel plot symmetry. The results still did not show any evidence of publication bias (All *P*>0.05). The funnel plot can be misleading [44], and Egger’s test may not really show publication bias [45]. To overcome these limitations, we performed the contour-enhanced funnel plot analyses to investigate the potential publication bias [46]. As shown in the Figure S1, no obvious publication bias was observed in the contrasts of Arg/Pro vs. Arg/Arg, Pro/Pro vs. Arg/Arg, dominant and recessive models, respectively.

## Discussion

In the present study, we collected all available, published studies and performed a meta-analysis to examine the association between the *p53* Arg72Pro polymorphism and susceptibility to hematological malignancies. Thirteen were critically reviewed to clarify controversial results from previous reports. Our meta-analysis showed that significantly increased NHL risks were found in all subjects with *p53* Arg72Pro polymorphism heterozygote and dominant model. No significant association was found between *p53* Arg72Pro polymorphism and leukemia risk.

Previous meta-analysis showed that *p53* Arg72Pro polymorphism was neither associated with hematological malignancies (eight studies), nor associated with leukemia risk (five studies) [11]. When stratified by ethnicities, a protective effect of the *p53* codon 72 Pro allele on leukemia was found in Asians even with a small number of studies (331 cases and 437 controls) [11]. With more studies and a larger number of subjects, our meta-analysis study confirmed that *p53* Arg72Pro polymorphism was not associated with hematological malignancies or leukemia risk. However, with more than double leukemia cases, we did not found an association between *p53* Arg72Pro polymorphism and leukemia risk in Asians (682 cases and 767 controls). We need to point that our meta-analysis study included all the cases from Takeuchi et al. study as leukemia patients, which may introduce bias since there were a few cases of lymphoma patients [42]. However, this did not affect the result when we excluded this study from this meta-analysis (Data not shown). Thus, our study cannot confirm the association between *p53* Arg72Pro polymorphism and leukemia risk in Asians and further studies with larger numbers of participants are needed to clarify this association.

On the other hand, the pathogenetic mechanisms of leukemia are different, and stratified analyses are required for different types of Leukemia. Due to the limited number of studies, this meta-analysis only performed subgroup analyses on the association between *p53* codon 72 Arg>Pro polymorphism and risk of AML (n = 5), and risk of other types of leukemia (n = 4) (Table 2). Future meta-analysis should analyze the association of genetic variants and different types of leukemia separately by including more emerging studies.

Recently, six studies were conducted to examine the association between *p53* Arg72Pro polymorphism and lymphoma risk [16], [17], [32], [33], [34], [36]. In the present meta-analysis, three studies were included [17], [32], [34], and the others were excluded due to the deviation from HWE [16], [33], [36]. Even including these three studies [16], [33], [36], the significant association was still found between *p53* Arg72Pro polymorphism and increased risk of all lymphoma (2,845 cases and 4,306 controls) (Arg/Pro *vs*. Arg/Arg: OR = 1.12, 95%CI: 1.01–1.25), and between *p53* Arg72Pro polymorphism and increased risk of NHL (2,547 cases and 4,306 controls) (Arg/Pro *vs*. Arg/Arg: OR = 1.11, 95%CI: 0.99–1.24) (Data not shown). This was consistent with our data that showed significant association between *p53* Arg72Pro polymorphism and increased NHL risk based on limited three studies. However, this meta-analysis has limitation by including indolent and aggressive lymphomas in the same group since pathogenetic mechanisms of different types of lymphomas are different. Therefore, additional well-designed large studies were required to validate the association between *p53* Arg72Pro polymorphism and increased risk of lymphomas.

The interaction of different polymorphisms in the same gene, or between different genes, might contribute to hematological malignancies risk. Although the combined effects of different *p53* polymorphisms have not been studied, the potential interactions between *p53* Arg72Pro polymorphism and other genetic polymorphisms, including those in *Murine double minute 2* (*MDM2*), *p73*, *p21* and *Glutathione S-transferase*, which are involved in DNA damage repair, apoptosis, cell cycle control, or detoxification of xenobiotic compounds, were found in hematological malignancies [15], [34], [35], [38], [43]. We evaluated the combined effects of these polymorphisms on susceptibility to hematological malignancies, however, due to the limited studies, the data were not sufficient to conduct a meta-analysis.

Heterogeneity for the *p53* Arg72Pro polymorphism was observed among these studies. The heterogeneity may be due to various factors, such as diversity in the population characteristics, differences in the number of cases and controls, genotyping methods and study design. Between-study heterogeneity was detected by restricted maximum likelihood-based random-effects meta-regression analysis. Because the number of included studies was limited, we conducted univariate meta-regression model firstly, variables with significant P values ≥0.1 were then entered into the multivariable model. Ethnicity, MAF, source of controls, sample size, publication years and disease types were taken into consideration, and none of these factors showed an evidence of source of heterogeneity (Table S3). To eliminate heterogeneity, we carried out subgroup analysis and used a random-effects model to pool the results whenever significant heterogeneity was present. In addition, some unpublished, eligible publications were not available in the present meta-analysis, which might affect the results.

In conclusion, we found significant associations between the *p53* Arg72Propolymorphism and lymphoma (non-Hodgkin lymphoma) risk, but not leukemia risk. However, the number of studies included for our meta-analysis is very limited, and studies based on larger well-designed populations are still needed to clarify the different effects of the *p53* Arg72Pro polymorphism in different types of hematological malignancies. Also, studies examining the combined effects of different *p53* polymorphisms or different polymorphisms of *p53* related genes (e.g., *MDM2*) should be investigated.

## Supporting Information

Figure S1 **Contour-enhanced funnel plot for publication bias analysis.**
(DOC)

Table S1 **Clinical and demographic characteristics of the patients in each study.**
(DOC)

Table S2 ***p53*** **Arg72Pro polymorphism genotype distribution of each study included in the meta-analysis.**
(DOC)

Table S3 **Meta-regression analysis.**
(DOC)
