# Supplementary material for: p53 codon 72 polymorphism and Hematological Cancer Risk: An Update Meta-Analysis
Source: PLoS One. 2012 Sep 24;7(9):e45820. doi: 10.1371/journal.pone.0045820 (PMC3454327; doi:10.1371/journal.pone.0045820)

**Supporting Information**

Figure S1 Contour-enhanced funnel plot for publication bias analysis. Contour-enhanced funnel plot for publication bias analysis on studies of p53 Arg72pro variant heterozygote (A), homozygote (B), dominant (C) and recessive model (D).


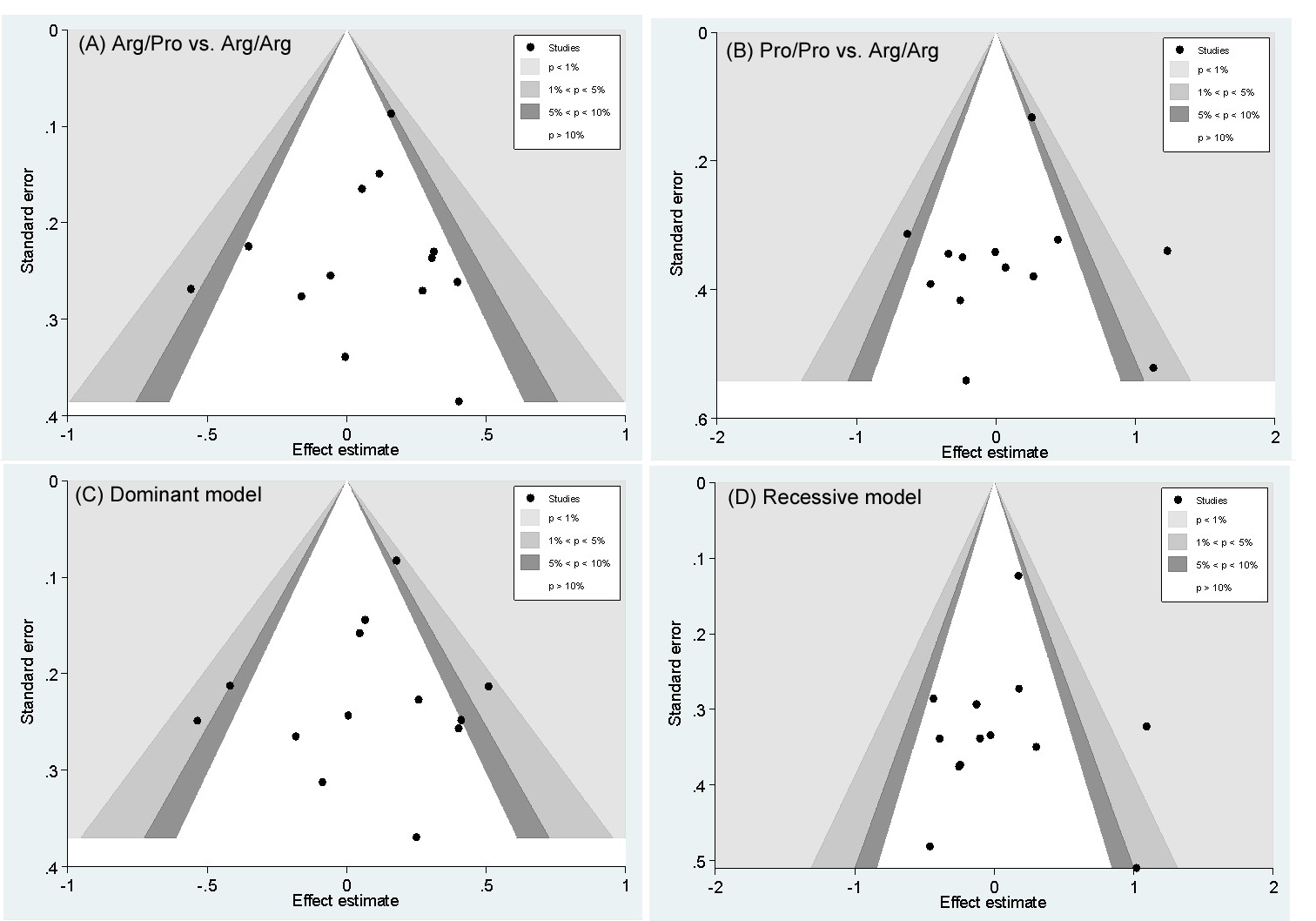

Supplement: Figure S1 — Contour-enhanced funnel plot for publication bias analysis. (DOC) [file pone.0045820.s001.doc]
