# Supplementary material for: p53 codon 72 polymorphism and Hematological Cancer Risk: An Update Meta-Analysis
Source: PLoS One. 2012 Sep 24;7(9):e45820. doi: 10.1371/journal.pone.0045820 (PMC3454327; doi:10.1371/journal.pone.0045820)
Supplement: Table S1 — Clinical and demographic characteristics of the patients in each study. (DOC) [file pone.0045820.s002.doc]

**Table S1 Clinical and demographic characteristics of the patients in each study.**

| Author | Year | Origin | Patients | Age | Gender | Disease |  |
| --- | --- | --- | --- | --- | --- | --- | --- |
| Leukemia |  |  |  |  |  |  |  |
| Nakano Y | 2000 | Japan | 200 | Adult | - | AML |  |
| Bergamaschi G | 2004 | Italy | 96 | - | - | CML |  |
| Takeuchi S | 2005 | Japan | 87 | Adult | - | ATL |  |
| Kochethu G | 2006 | UK | 203 | 68 | F (116), M (87) | CLL |  |
| Phang BH | 2008 | China | 44 | 44 | F (22), M (22) | AML (26) BAL (5) ALL (13) |  |
| Ellis NA | 2008 | USA/UK | 171 | 56 | F (96), M (75) | AML |  |
| Xiong X | 2009 | China | 231 | 35 | F (83), M (148) | AML |  |
| Do TN | 2009 | US | 114 | Child | - | ALL |  |
| Chauhan PS | 2011 | India | 120 | 36 | F (43), M (77) | AML |  |
| Lymphomas |  |  |  |  |  |  |  |
| Hishida A | 2004 | Japan | 103 | 53 | F (46), M (57) | NHL |  |
| Bittenbring J | 2008 | Germany | 311 | 62 | F (136), M(175) | NHL |  |
| Kim HN | 2010 | Korea | 945 | 58 | - | NHL |  |
| Myeloma |  |  |  |  |  |  |  |
| Ortega MM | 2007 | Brazil | 106 | 60 | F (48), M(58) | MM |  |

Abbreviations: ALL, Acute lymphocytic leukemias; AML, Acute myelogenous leukemia; BAL, Biphenotypic acute leukaemia; CLL, Chronic lymphocytic leukemias; CML, Chronic myelogenous leukemia; ATL, Adult T-cell leukaemia/lymphoma; MM, Multiple myeloma; NHL, non-Hodgkin lymphoma
