# Supplementary material for: p53 codon 72 polymorphism and Hematological Cancer Risk: An Update Meta-Analysis
Source: PLoS One. 2012 Sep 24;7(9):e45820. doi: 10.1371/journal.pone.0045820 (PMC3454327; doi:10.1371/journal.pone.0045820)
Supplement: Table S2 — p53 Arg72Pro polymorphism genotype distribution of each study included in the meta-analysis. (DOC) [file pone.0045820.s003.doc]

**Table S2 *p53* Arg72Pro polymorphism genotype distribution of each study included in the meta-analysis**

| Author | Year | Case | | | Control | | | | |
| --- | --- | --- | --- | --- | --- | --- | --- | --- | --- |
|  |  | Arg/Arg | Arg/Pro | Pro/Pro | Arg/Arg | | Arg/Pro | Pro/Pro |  |
| Leukemia |  |  |  |  | |  |  |  | |
| Nakano Y | 2000 | 82 | 93 | 25 | | 59 | 95 | 34 | |
| Bergamaschi G | 2004 | 49 | 37 | 10 | | 106 | 61 | 7 | |
| Takeuchi S | 2005 | 33 | 38 | 16 | | 32 | 37 | 20 | |
| Kochethu G | 2006 | 119 | 62 | 22 | | 44 | 40 | 13 | |
| Phang BH | 2008 | 13 | 25 | 6 | | 56 | 72 | 32 | |
| Ellis NA | 2008 | 95 | 66 | 10 | | 1714 | 1127 | 181 | |
| Xiong X | 2009 | 52 | 127 | 52 | | 39 | 64 | 25 | |
| Do TN | 2009 | 50 | 45 | 19 | | 234 | 154 | 26 | |
| Chauhan PS | 2011 | 32 | 66 | 22 | | 47 | 114 | 41 | |
| Lymphomas |  |  |  |  | |  |  |  | |
| Hishida A | 2004 | 37 | 54 | 12 | | 185 | 199 | 56 | |
| Bittenbring J | 2008 | 168 | 130 | 13 | | 285 | 196 | 31 | |
| Kim HN | 2010 | 367 | 455 | 123 | | 734 | 776 | 190 | |
| Myeloma |  |  |  |  | |  |  |  | |
| Ortega MM | 2007 | 39 | 52 | 15 | | 85 | 120 | 25 | |
