# Supplementary material for: p53 codon 72 polymorphism and Hematological Cancer Risk: An Update Meta-Analysis
Source: PLoS One. 2012 Sep 24;7(9):e45820. doi: 10.1371/journal.pone.0045820 (PMC3454327; doi:10.1371/journal.pone.0045820)
Supplement: Table S3 — Meta-regression analysis. (DOC) [file pone.0045820.s004.doc]

| **Table S3 Meta-regression analysis** | | | | | | | |
| --- | --- | --- | --- | --- | --- | --- | --- |
| Contrast | Univariable model | | | | | | Multivariable model |
|  | Ethnicity | MAF | Source | Size | Year | Disease |  |
| Arg/Pro vs. Arg/Arg | 0.716 | 0.383 | 0.440 | 0.628 | 0.157 | 0.337 | - |
| Pro/Pro vs. Arg/Arg | 0.329 | 0.122 | 0.837 | 0.873 | 0.271 | 0.969 | - |
| Dominant | 0.893 | 0.222 | 0.604 | 0.734 | 0.179 | 0.611 | - |
| Recessive | 0.199 | 0.111 | 0.607 | 0.878 | 0.333 | 0.884 | - |
